# Finger Lacerations Are Highly Prevalent Among Shoulder Surgeons, and No Widely Effective Preventive Measures Are Available

**DOI:** 10.1002/ars2.70138

**Published:** 2026-11-10

**Authors:** Anh Do, Asimina Lazaridou, Markus Scheibel

**Affiliations:** ^1^ Center for Musculoskeletal Surgery Charité University Hospital Berlin Germany; ^2^ Schulthess Klinik Zurich Switzerland

## Abstract

**Purpose:**

To explore factors associated with the occurrence of finger lacerations during arthroscopic and open knot‐tying techniques in shoulder surgery.

**Methods:**

This cross‐sectional survey was conducted at an international shoulder surgery course in March 2024. Attending shoulder surgeons performing arthroscopic and open shoulder surgeries were asked to participate in a survey on site using a structured questionnaire to explore factors associated with the occurrence and anatomical distribution of skin lacerations during arthroscopic and open knot‐tying techniques. Demographic, procedural, and intraoperative variables were collected and compared between techniques. Linear and logistic regression analyses were performed to identify predictors of laceration occurrence and severity.

**Results:**

A total of 100 surgeons were invited to participate, all of whom completed the survey (response rate: 100%). Eighty‐nine percent of respondents reported having experienced finger lacerations either currently or in the past, with 79% still affected at the time of the survey. The remaining 10% had adopted effective preventive measures: 3% modified their knot‐tying technique, 2% wrapped tape around the affected fingers, and 5% developed hyperkeratosis as a protective adaptation. Among those affected, lacerations were reported to occur always in 20%, often in 42%, sometimes in 22%, and rarely in 16% of arthroscopic cases. The distal interphalangeal joints of the index and little fingers were most commonly involved, with the index finger affected in 71%, the little finger in 30%, and 18% reporting lesions on both fingers. Rotator cuff reconstruction was the procedure most frequently associated with laceration occurrence; however, no significant correlation was found between laceration frequency and the number of reconstructions performed per year (*r* = 0.091, *P* = .371). Similarly, no association was observed between handedness and the affected side, with 43% reporting bilateral involvement (*χ* ^2^ = 2.464, *P* = .292). Hyperkeratosis was observed in 54.4% of affected surgeons, suggesting a potential adaptive response to repetitive skin trauma.

**Conclusions:**

Finger lacerations are a common problem among shoulder surgeons. Seventy‐nine percent of respondents reported being regularly affected, particularly at the distal interphalangeal joint of the index and little fingers. Surgeons performing rotator cuff repairs showed a significantly higher incidence of finger lacerations at present.

**Clinical Relevance:**

Finger lacerations and glove microlesions during knot tying are common among shoulder surgeons and may compromise barrier integrity. The lack of widely effective preventive measures underscores the need for improved strategies to protect both surgeons and patients.

## Graphical abstract

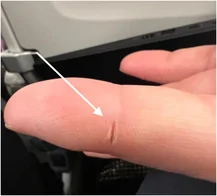

Knot tying represents a critical technical step in most arthroscopic and open shoulder procedures, ensuring secure fixation and repair integrity. However, it frequently results in finger lacerations (Figure 1) and microlesions in surgical gloves, which may compromise their protective function and serve as a potential source of infection for both surgeon and patient.^1^, ^2^, ^3^, ^4^ Recent studies have shown that such microlesions allow bacterial passage and epithelial cell transfer, providing a potential source of contamination, whereas the skin lacerations resulting from knot tying may serve as an entry point for pathogens.^1^, ^5^

**FIGURE 1:**
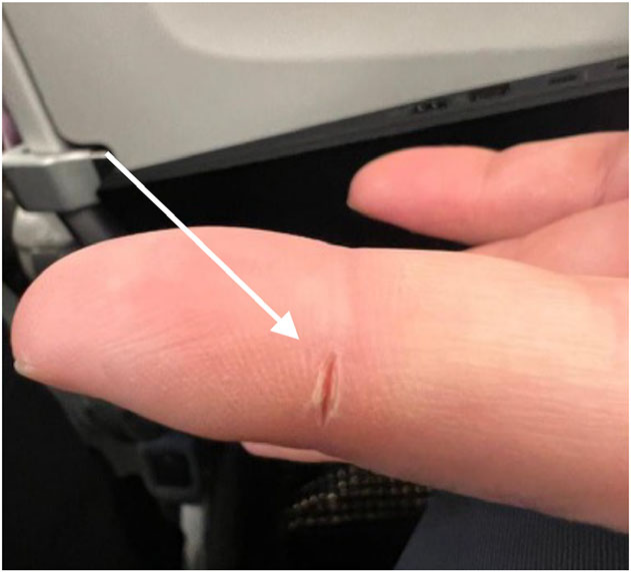
Skin laceration over the distal interphalangeal joint of the surgeon's left index finger (white arrow).

Several preventive measures, such as finger taping, have been proposed to prevent the occurrence of finger lacerations; however, none have proven widely effective. To develop effective preventive strategies and ensure the protection of both patients and surgeons, analyzing the influencing factors and anatomical locations of skin lacerations is of great importance. The development of such strategies may help reduce the risk of infection.

The purpose of this study was to explore factors associated with the occurrence of finger lacerations during arthroscopic and open knot‐tying techniques in shoulder surgery. The hypothesis was that many surgeons are affected by finger lacerations and no widely used preventative measures currently exist.

## METHODS

This cross‐sectional survey was conducted at an international shoulder surgery course in March 2024. Attending shoulder surgeons performing arthroscopic and open shoulder surgeries were asked to participate in a survey on site using a structured questionnaire (Supporting Information) to collect data on handedness and surgical activity, including the number and type of procedures performed, suture materials and number of gloves used for each procedure, and the knot‐tying technique used. To assess finger lacerations, information was documented on their anatomical location, self‐reported frequency of occurrence (always, often, sometimes, rarely, and never), presence of hyperkeratosis, and any preventive measures applied. The study was reviewed and approved by the local ethics committee of Charité Universitätsmedizin (EA4/107/24), and all participants provided informed consent to participate in the study.

### Statistical Analysis

Statistical analysis was performed with IBM SPSS Statistics software (Version 29; IBM), and a value of *P* < .05 was considered statistically significant. The Kolmogorov‐Smirnov test was used to test for normal distribution. Categorical variables were reported as frequency and percentage. Continuous variables were presented as mean ± standard deviation when normally distributed or median (Q1‐Q3 interquartile range) when non‐normally distributed. For dichotomous data, the *χ* ^2^ test was used. The correlation between continuous variables and laceration frequency was assessed using the Spearman correlation coefficient. Binary logistic regression was performed to evaluate the influence of surgery frequency on the occurrence of finger lacerations and hyperkeratosis and to calculate odds ratios with 95% confidence intervals. In addition, logistic and linear regression analyses were used to further explore associations between procedural variables and outcome measures.

## RESULTS

A total of 100 surgeons were invited to participate, all of whom completed the survey (response rate: 100%), including 94% men and 6% women. The respondent characteristics are shown in Table 1.

**TABLE 1:** Respondent Characteristics

**Variable**	**n = 100**
Gender	100
Men, n (%)	94 (94%)
Women, n (%)	6 (6%)
Handedness	45 (27.5%)
Right, n (%)	88 (88%)
Left, n (%)	12 (12%)
Surgeries per year, median (IQR)	300 (200‐469)
Rotator cuff repairs	100 (40‐150)
Bankart repairs	20 (5‐49)
Shoulder arthroplasties	48 (20‐80)
ORIF of proximal humeral fractures	22 (10‐50)
Surgery frequency per week, median (IQR)	
Rotator cuff repairs	2.5 (1‐4)
Bankart repairs	0.5 (0.1‐1)
Shoulder arthroplasties	1 (0.5‐2)
ORIF of proximal humeral fractures	0.5 (0.3‐1)
Number of gloves	
Single gloving for all procedures, n (%)	7 (7%)
Double gloving for all procedures, n (%)	86 (86%)
Single gloving for arthroscopic procedures	
Double gloving for open procedures, n (%)	7 (7%)

IQR, interquartile range; ORIF, Open Reduction and Internal Fixation.

### Finger Lacerations

At the time of the survey, 89% of the respondents reported having experienced finger lacerations during knot tying, either currently or in the past, with 79% still affected at present. The remaining 10% reported measures they considered effective in reducing lacerations: 3% modified their knot‐tying technique, 2% used finger taping, and 5% reported hyperkeratosis at the affected sites. Hyperkeratosis may offer partial protection against finger lacerations but represents a reactive adaptation rather than a deliberate preventive measure. Additionally, 32% of respondents reported attempting preventive measures without success, including 5% with hyperkeratosis, 8% with technique modifications, and 19% with finger taping.

Among those affected, skin lacerations occurred always in 20% of cases, often in 42%, sometimes in 22%, and rarely in 16% (Figure 2). The index and little fingers were most frequently affected, with the index finger involved in 71% of surgeons and the little finger in 30%, including 18% with lesions on both fingers (Figure 3, Table 2). The most commonly affected sites were the distal interphalangeal joints of the index finger (58% of surgeons) and little finger (26% of respondents) (Figure 4).

**FIGURE 2:**
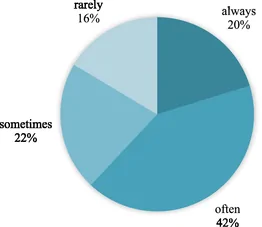
Pie chart illustrating the frequency of finger lacerations among affected respondents.

**FIGURE 3:**
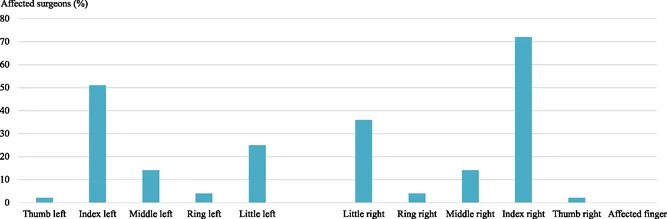
Bar chart illustrating the distribution of finger lacerations across different fingers.

**TABLE 2:** Distribution of Affected Fingers Among Shoulder Surgeons

**Finger**	**Surgeons Affected in %**
Thumb	3%
Index finger	71%
Middle finger	16%
Ring finger	7%
Little finger	30%

**FIGURE 4:**
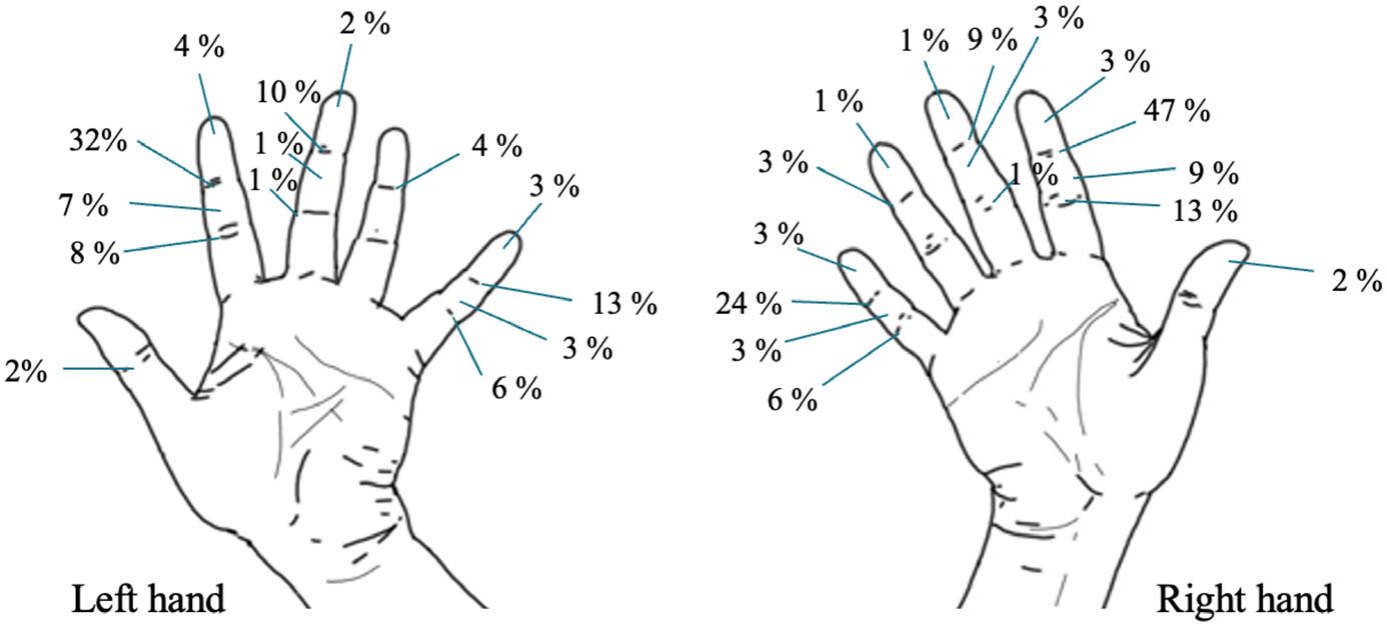
Representation of the distribution of finger lacerations on the left and right hand. Percentages represent the proportion of affected respondents.

Rotator cuff reconstruction was most frequently reported as the cause of the lacerations. Surgeons who perform rotator cuff repairs showed a significantly higher incidence of finger lacerations at present (77/94, 81.9%) compared with those who do not perform this surgery (2/6, 33.3%) (*χ* ^2^ = 8.024, *P* = .005). However, no association was found between the frequency of finger lacerations and the number of rotator cuff reconstructions performed per year (*r* = 0.091, *P* = .371). Similarly, no correlation was observed between the frequency of finger lacerations and the number of Bankart repairs, shoulder arthroplasties, and ORIF of proximal humeral fractures per year (Table 3).

**TABLE 3:** Correlation Between Surgical Volume and Laceration Frequency∗

**Variable**	***r***	***P*** **Value**
Rotator cuff repairs per year	0.019	.371
Bankart repairs per year	0.177	.79
Shoulder arthroplasties per year	0.024	.819
ORIF of proximal humeral fractures per year	0.178	.094

ORIF, Open Reduction and Internal Fixation.

∗ Spearman correlation coefficients (*r*) are reported.

Among surgeons performing rotator cuff repairs, 9.6% used knotted anchors and 90.4% used knotless anchors, with no significant difference in finger laceration rates between groups (*P* = .568). Of those using knotted anchors, 89.4% used sliding knots, and 10.6% used nonsliding knots, with no significant difference in finger laceration rates between groups (*P* = .783). No correlation was found between the laceration rates and the number of additional half‐hitches (*P* = .268). Moreover, hyperkeratosis was significantly more common among surgeons who currently experienced finger lacerations (43/79, 54.4%) compared with those without lacerations (6/21, 28.6%) (*χ* ^2^ = 4.439, *P* = .035).

The proportion of respondents reporting finger lacerations was slightly higher among surgeons with single gloving during arthroscopic procedures (12/14, 85.7%) compared with surgeons with double gloving (67/86, 77.9%), although this difference was not statistically significant (*χ* ^2^ = 0.442, *P* = .506). Furthermore, no association was found between handedness and the affected side (*χ* ^2^ = 2.464, *P* = .292) because 43% reported lacerations on both hands. Logistic regression analysis revealed that a higher weekly frequency of rotator cuff repairs (odds ratio, 1.253; 95% confidence interval, 1.059‐1.482; *P* = .009) and Bankart repairs (odds ratio, 2.160; 95% confidence interval, 1.183‐3.942; *P* = .012) were both associated with an increased risk of developing hyperkeratosis (Table 4).

**TABLE 4:** Analysis of the Influence of Surgery Frequency on the Occurrence of Hyperkeratosis

**Surgery Frequency per Week**	**Median (IQR)**	**OR**	**95% CI**	***P*** **Value**
Rotator cuff repairs	2.5 (1‐4)	1.253	1.059‐1.482	**.009**
Bankart repairs	0.5 (0.1‐1)	2.160	1.183‐3.942	**.012**
Shoulder arthroplasties	1 (0.5‐2)	1.245	0.936‐1.658	.132
ORIF of proximal humeral fractures	0.5 (0.3‐1)	0.795	0.546‐1.158	.232

*Note*: Bold *P* values are statistically significant (*P* < .05).

CI, confidence interval; IQR, interquartile range; OR, odds ratio; ORIF, Open Reduction and Internal Fixation.

## DISCUSSION

Finger lacerations during arthroscopic and open knot tying are a common problem among shoulder surgeons, affecting 79% of respondents. Ten percent reported lacerations in the past but have developed effective coping mechanisms to prevent them. Different fingers are affected, most commonly the index finger (71% of respondents), followed by the little finger (30%) and the middle finger (16%), with the distal interphalangeal joints of the index and little fingers as the most frequently affected sites. Surgeons who perform rotator cuff repairs showed a significantly higher incidence of finger lacerations at present.

Four studies have reported on glove lesions and finger lacerations during knot tying.^1^, ^2^, ^3^, ^4^ Enz et al.^1^ evaluated the impact of surgical knot tying on glove integrity and reported glove damage in 25% of rotator cuff repair surgeries. They conducted bacterial pass‐through tests on the glove lesions and found *Staphylococcus hominis* and *Bacillus cereus*, representing likely scenarios of contamination during surgery. In their study, skin lacerations resulting from knot tying were described as potential entry points for pathogens; however, the exact anatomical locations of these finger lacerations were not specified. Furthermore, no previous study has reported on the influencing factors and anatomical locations of skin lacerations resulting from knot tying in a larger cohort of shoulder surgeons.

In a study by Burn et al.,^5^ epithelial cells were transmitted to the suture during simulated arthroscopic knot tying. However, the number of epithelial cells did not correlate with the number of knots tied or the presence of glove tears, despite the occurrence of skin lacerations during knot tying. It should be noted that although skin keratinocytes vary between 15 and 35 μm in size, pathogens such as human immunodeficiency and hepatitis viruses are much smaller, between 0.1 and 0.042 μm in size, and could therefore serve as a source of contamination. In their study, the finger lacerations occurred on the radial side of the left index finger in the 2 performing surgeons.

Kaplan et al.^2^ revealed that arthroscopic knot tying with solid‐core sutures frequently results in intraoperative glove tears and associated finger lacerations. Finger lacerations were also observed even when gloves remained intact. Nevertheless, the presence of an inner glove tear was significantly associated with lacerations at the corresponding finger level. In their study, many surgeons reported hyperkeratosis at the distal interphalangeal joint from previous finger lacerations, consistent with our findings.

In contrast, in an experimental study by Giordano et al.,^3^ no damage to the surgical gloves was observed during the experiment. Skin lacerations were detected on the ulnar side of the little finger in both surgeons’ hands, likely caused by friction. Despite the common occurrence of these lacerations, no preventive measures have proven widely effective among affected surgeons.

### Limitations

This study is subject to several limitations, including recall and response bias, because data were based on self‐reported information (e.g., finger laceration frequency) and not all data could be verified. The high variability in suture materials, gloves, and knot‐tying techniques among surgeons, as well as the use of knotless anchors by some, may have influenced the assessment of factors associated with finger lacerations. Additionally, unmeasured factors such as surgeon experience, suture characteristics, knot‐tying speed, glove use, and intraoperative ergonomics may also have affected the risk of finger lacerations, introducing potential confounders. Furthermore, the proportion of female participants in our cohort was low (6%), which precluded meaningful sex‐based comparisons.

## CONCLUSIONS

Finger lacerations are a common problem among shoulder surgeons. Seventy‐nine percent of respondents reported being regularly affected, particularly at the distal interphalangeal joint of the index and little fingers. Surgeons performing rotator cuff repairs showed a significantly higher incidence of finger lacerations at present.

## SUPPORTING INFORMATION

Additional supporting information can be found online in the Supporting Information section.

## DISCLOSURES

The authors (A.D., A.L., M.S.) declare that they have no known competing financial interests or personal relationships that could have appeared to influence the work reported in this paper.

## Supporting information

Supplementary Material

## References

[1] Enz A , Klinder A , Bisping L , et al. Knot tying in arthroplasty and arthroscopy causes lesions to surgical gloves: A potential risk of infection. Knee Surg Sports Traumatol Arthrosc. 2023;31:1824‐1832. 10.1007/s00167-022-07136-7 PMC10089991 36048202

[2] Kaplan KM , Gruson KI , Gorczynksi CT , Strauss EJ , Kummer FJ , Rokito AS . Glove tears during arthroscopic shoulder surgery using solid‐core suture. Arthroscopy. 2007;23:51‐56. 10.1016/j.arthro.2006.10.012 17210427

[3] Giordano V , Koch HA , de Sousa Prado J , et al. Is the surgical knot tying technique associated with a risk for unnoticed glove perforation? An experimental study. Patient Saf Surg. 2014;8:26. 10.1186/1754-9493-8-26 PMC4078929 24991234

[4] Martinez A , Han Y , Sardar ZM , et al. Risk of glove perforation with arthroscopic knot tying using different surgical gloves and high‐tensile strength sutures. Arthroscopy. 2013;29:1552‐1558. 10.1016/j.arthro.2013.05.022 23876607

[5] Burn MB , Holtorf HL , Smith KM , et al. Do skin lacerations imply tissue transfer from surgeon to patient during arthroscopic knot tying? Arthroscopy. 2017;33:2248‐2254. 10.1016/j.arthro.2017.07.033 29066268

