# Supplementary material for: Finger Lacerations Are Highly Prevalent Among Shoulder Surgeons, and No Widely Effective Preventive Measures Are Available
Source: Arthrosc Sports Med Rehabil. 2026 Nov 10:e70138. Online ahead of print. doi: 10.1002/ars2.70138 (PMC13749472; doi:10.1002/ars2.70138)
Supplement: Supplementary file 1 [file ARS2-9999-e70138-s001.pdf]

Survey Lacerations

<table>
<tr><td>Full name</td><td colspan="4"></td></tr>
<tr><td>Contact details</td><td colspan="4"></td></tr>
<tr><td>Handedness</td><td colspan="4">o right o left</td></tr>
<tr><td>Surgeries per year</td><td colspan="4"></td></tr>
<tr><td>Surgeries</td><td colspan="4">Shoulder: o Arthoscopy o Arthroplasty o Trauma
other:</td></tr>
<tr><td>Surgeries with knots</td><td>RCR</td><td>Stabilization</td><td>Arthroplasty</td><td>Fractures</td></tr>
<tr><td>Number per year:</td><td></td><td></td><td></td><td></td></tr>
<tr><td>Frequency:</td><td>/week</td><td>/week</td><td>/week</td><td>/week</td></tr>
<tr><td>Anchor/Suture:</td><td></td><td></td><td></td><td></td></tr>
<tr><td>Knot technique:</td><td>o knot pusher</td><td>o knot pusher</td><td></td><td></td></tr>
<tr><td>Hand lacerations and hyperkeratosis</td><td colspan="2">Frequency of hand lacerations:

o Always
o Often
o Sometimes
o Rarely
o Never

Hyperkeratosis

o yes
o no</td><td colspan="2">Locations of lacerations x

Left hand Right hand
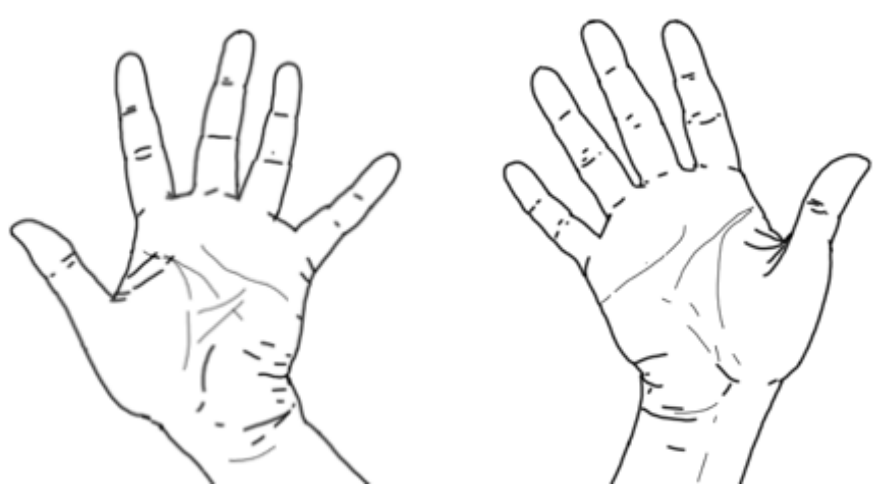

Surgeries causing lacerations:
.</td></tr>
<tr><td>Laceration prevention</td><td colspan="4"></td></tr>
<tr><td>Glove brand and model</td><td colspan="4"></td></tr>
<tr><td>Number of glove pairs</td><td colspan="4">o Double gloving o Double glove with indicator o Single gloving</td></tr>
</table>
